# Orthohantaviruses in Reservoir and Atypical Hosts in the Czech Republic: Spillover Infection and Indication of Virus-Specific Tissue Tropism

**DOI:** 10.1128/spectrum.01306-22

**Published:** 2022-09-28

**Authors:** Václav Hönig, Jan Kamiš, Aneta Maršíková, Tereza Matějková, Pavel Stopka, Anna Mácová, Daniel Růžek, Jana Kvičerová

**Affiliations:** a Laboratory of Arbovirology, Institute of Parasitology, Biology Centre CAS, České Budějovice, Czech Republic; b Laboratory of Emerging Viral Infections, Veterinary Research Institute, Brno, Czech Republic; c Department of Parasitology, Faculty of Science, University of South Bohemia, České Budějovice, Czech Republic; d Department of Zoology, Faculty of Science, Charles University, Biocev, Vestec, Czech Republic; e Department of Experimental Biology, Faculty of Science, Masaryk University, Brno, Czech Republic; Erasmus MC

**Keywords:** Kurkino virus, Tula virus, Seewis virus, Asikkala virus, rodents, Eulipotyphla, phylogeny, host specificity, tissue specificity, zoonoses, zoonosis

## Abstract

Orthohantaviruses (genus *Orthohantavirus*) are a diverse group of viruses that are closely associated with their natural hosts (rodents, shrews, and moles). Several orthohantaviruses cause severe disease in humans. Central and western Europe are areas with emerging orthohantavirus occurrences. In our study, several orthohantaviruses, including the pathogenic Kurkino virus (KURV), were detected in their natural hosts trapped at several study sites in the Czech Republic. KURV was detected mainly in its typical host, the striped field mouse (Apodemus agrarius). Nevertheless, spillover infections were also detected in wood mice (Apodemus sylvaticus) and common voles (Microtus arvalis). Similarly, Tula virus (TULV) was found primarily in common voles, and events of spillover to rodents of other host species, including *Apodemus* spp., were recorded. In addition, unlike most previous studies, different tissues were sampled and compared to assess their suitability for orthohantavirus screening and possible tissue tropism. Our data suggest possible virus-specific tissue tropism in rodent hosts. TULV was most commonly detected in the lung tissue, whereas KURV was more common in the liver, spleen, and brain. Moreover, Seewis and Asikkala viruses were detected in randomly found common shrews (Sorex araneus). In conclusion, we have demonstrated the presence of human-pathogenic KURV and the potentially pathogenic TULV in their typical hosts as well as their spillover to atypical host species belonging to another family. Furthermore, we suggest the possibility of virus-specific tissue tropism of orthohantaviruses in their natural hosts.

**IMPORTANCE** Orthohantaviruses (genus *Orthohantavirus*, family *Hantaviridae*) are a diverse group of globally distributed viruses that are closely associated with their natural hosts. Some orthohantaviruses are capable of infecting humans and causing severe disease. Orthohantaviruses are considered emerging pathogens due to their ever-increasing diversity and increasing numbers of disease cases. We report the detection of four different orthohantaviruses in rodents and shrews in the Czech Republic. Most viruses were found in their typical hosts, Kurkino virus (KURV) in striped field mice (Apodemus agrarius), Tula virus (TULV) in common voles (*Microtus arvalis*), and Seewis virus in common shrews (*Sorex araneus*). Nevertheless, spillover infections of atypical host species were also recorded for KURV, TULV, and another shrew-borne orthohantavirus, Asikkala virus. In addition, indications of virus-specific patterns of tissue tropism were observed. Our results highlight the circulation of several orthohantaviruses, including KURV, which is pathogenic to humans, among rodents and shrews in the Czech Republic.

## INTRODUCTION

Orthohantaviruses (genus *Orthohantavirus*, family *Hantaviridae*, order *Bunyavirales*) are negative-sense, enveloped, single-stranded zoonotic RNA viruses with a trisegmented genome (formed by large [L], medium [M], and small [S] segments) (1, 2). In humans, they may cause infection with two types of clinical manifestations, both with possibly fatal outcomes (3, 4). Hemorrhagic fever with renal syndrome (HFRS) is caused by Old World orthohantaviruses that occur in Europe and Asia, whereas hantavirus pulmonary (or cardiopulmonary) syndrome [H(C)PS] is caused by New World orthohantaviruses in the Americas (5, 6). Orthohantaviruses are considered host specific and are tightly associated with hosts of one or a few closely related species that constitute their natural reservoir (6–9). The reservoir hosts of orthohantaviruses that are pathogenic to humans are rodents, but other orthohantaviruses have also been detected in Eulipotyphla (namely, shrews and moles) (10, 11). As rodents are widespread and people can easily come into contact with them, human infections have become an increasing problem. The inhalation of virus-containing aerosols via the excreta (urine, feces, or saliva) of infected rodents is the most common route of transmission (10, 12).

In general, orthohantaviruses form three large evolutionary groups (see below) associated with hosts from four rodent subfamilies, including the Old World subfamilies Murinae (family Muridae) and Arvicolinae (family Cricetidae) and the New World subfamilies Sigmodontinae (Cricetidae) and Neotominae (Cricetidae) (8, 13). In addition, some orthohantaviruses are associated with hosts of the order Eulipotyphla (families Soricidae and Talpidae) as their reservoir hosts (13). In Europe, the following orthohantaviruses circulate in populations of wild rodents: Dobrava virus (DOBV), Kurkino virus (KURV), Saaremaa virus (SAAV), Sochi virus (SOCV) (all belonging to the *Dobrava-Belgrade orthohantavirus* species), Puumala virus (PUUV) (*Puumala orthohantavirus*), Seoul virus (SEOV) (*Seoul orthohantavirus*), and Tula virus (TULV) (*Tula orthohantavirus*) (8, 14–17). Moreover, Seewis virus (SWSV) (*Seewis orthohantavirus*) and Asikkala virus (ASIV) (*Asikkala orthohantavirus*) have been found mainly in shrews (18, 19). Most of the European orthohantavirus human disease cases are caused by PUUV, DOBV, and KURV (20). The viruses differ in their geographic distributions, species of reservoir hosts, and virulence to humans. DOBV (previously known as DOBV-Af), typically hosted by yellow-necked mice (Apodemus flavicollis; Murinae), is dominant in the Balkans and Russia (21). It has also been found in several countries in central Europe (e.g., the Czech Republic, Germany, Hungary, and Slovakia) (8, 21, 22). KURV (previously known as DOBV-Aa) is associated with striped field mice (Apodemus agrarius), is widely distributed from Germany throughout the central European countries to parts of northern (Denmark) and eastern (Estonia and Russia) Europe, and causes a milder form of human disease than DOBV (8, 23, 24). Striped field mice are also the reservoir hosts of SAAV, so far restricted to the island of Saaremaa in Estonia (7). SOCV (previously known as DOBV-Ap) is associated with Black Sea field mice (Apodemus ponticus) and occurs in the Black Sea region of the European part of Russia (7, 25). The more common but less virulent PUUV is the causative agent of an HFRS-like disease called nephropathia epidemica (NE) (3). Together with its reservoir host, the bank vole (Clethrionomys glareolus; Arvicolinae), it is distributed throughout Europe and in the western part of Russia (23, 26). Furthermore, the cooccurrence of PUUV, DOBV, and KURV in the same area has been reported, particularly in the Balkans (27). SEOV, which is transmitted by rats (*Rattus* spp.; Murinae), is an exceptional orthohantavirus that is distributed worldwide due to ship trade and human migration, allowing the movement of rats over long distances (26, 28). TULV is found primarily in common voles (Microtus arvalis; Arvicolinae), several other members of the same genus, and European water voles (Arvicola amphibius; Arvicolinae) (29–31). Although TULV is considered nonpathogenic, rare cases of TULV-associated pulmonary and renal syndrome have been documented in humans in the Czech Republic and Germany (32, 33).

Regarding shrew-borne orthohantaviruses, SWSV was first detected in a common shrew (Sorex araneus; Soricidae) captured in a Swiss village of the same name (34). Since then, several studies have confirmed SWSV in shrews and also occasionally in rodents in other central European countries, including the Czech Republic, Slovakia, and Germany (19, 35). Another shrew-borne hantavirus, ASIV, has been recorded as a novel hantavirus from Finland (36), carried by the Eurasian pygmy shrew (Sorex minutus). Together with SWSV, ASIV has also been detected in the Czech Republic and neighboring Germany (18).

Although orthohantaviruses are not new to humankind, they are considered to be emerging viruses with epidemic outbreaks because of the recent increase in the number of human cases (especially in western Europe) (37) and because of the continuous records of enormous previously unrecognized diversity (5, 7, 38, 39). In contrast to the observed seroprevalence (22), the incidence of orthohantavirus infection in humans is lower in the Czech Republic than in neighboring Germany or Austria (20, 40). Data on the circulation of orthohantaviruses among reservoir hosts are incomplete, yet human cases and rodent tissue screening suggest the presence and epidemiologic relevance of DOBV, KURV, PUUV, and TULV (35) in this country. Here, we report KURV and TULV, their phylogenetic relationships, and their occurrence in different host tissues of wild rodents mainly from urban areas of the Czech Republic as well as SWSV and ASIV in randomly found shrews.

## RESULTS

Altogether, 153 rodent individuals were trapped and sampled at the defined trapping sites (for details, see Table 1). Moreover, 10 randomly found dead shrews (family Soricidae; *Sorex* spp., *Crocidura* spp., and Neomys fodiens) were also sampled (Table 2).

**TABLE 1:** Summary of the numbers and species of the trapped and examined rodents in the Czech Republic from 2016 to 2021

Locality (region)	Trapping yr(s)	No. of rodents of species				
		*Microtus arvalis*	*Clethrionomys glareolus*	Apodemus agrarius	*Apodemus sylvaticus*	*Apodemus flavicollis*
České Budějovice (South Bohemia)	2016–2018	4	7		12	15
Lužnice (South Bohemia)	2018		10			1
Zbytiny-Koryto (South Bohemia)	2021		2		5	
Květušín (South Bohemia)	2021	2			1	2
Opava (Northern Moravia)	2016	1		40	1	10
Varnsdorf (Northern Bohemia)	2018, 2019	1	1	6		1
Vestec (Central Bohemia)	2020	16			6	9

Total		24	20	46	25	38

**TABLE 2:** Detailed information on the randomly found dead shrews

Locality ID	Name of locality (district)	Locality type	GPS coordinates (WGS84)	Yr of collection	Species of collected animal
A	České Budějovice, Vltava (České Budějovice)	Urban area (housing estate)	48°59′56.238″N, 14°27′19.339″E	2017	*Sorex minutus*
B	České Budějovice, Biology Centre CAS (České Budějovice)	Urban area (research center complex)	48°58′39.859″N, 14°26′52.175″E	2020	*Sorex araneus*
C	Zbytiny-Koryto (Prachatice)	Area of confirmed hantavirus disease in humans	48°55′53.899″N, 14°01′23.761″E	2018	*Sorex araneus*
D	Volenice (Strakonice)	Rural area (agricultural)	49°32′26.700″N, 13°54′06.000″E	2019	*Crocidura suaveolens*
E	Lužnice, field station U Zahradníků no. 92 (Jindřichův Hradec)	Rural area (congress center)	49°04′51.428″N, 14°45′41.266″E	2018	*Neomys fodiens* (*n* = 2)
F	Hoděmyšl (Příbram)	Urban area	49°36′41.220″N, 13°53′17.700″E	2019	*Crocidura suaveolens*
G	Podmokly (Plzeň-sever)	Rural area (agricultural)	49°52′04.020″N, 13°10′00.240″E	2019	*Sorex araneus*
H	Varnsdorf (Děčín)	Rural area (agricultural)	50°55′09.899″N, 14°35′53.808″E	2018	*Sorex araneus*
I	Semtěš (Karlovy Vary)	Rural area (agricultural)	50°04′32.460″N, 13°09′41.700″E	2019	*Crocidura leucodon*

### Prevalence and diversity of the detected orthohantaviruses.

In total, 24.2% (37/153) of the rodent hosts and 27.3% (3/10) of the shrews tested positive for orthohantavirus RNA (PCR products confirmed by sequencing) in at least one tissue sample (multiple tissue samples were taken from a trapped individual). Based on nucleotide sequence analysis, TULV, KURV, SWSV, and ASIV were identified in the positive samples. TULV was most frequently found in common voles (70.8% of all trapped common voles), and KURV was most frequently found in striped field mice (15.2% of all trapped striped field mice), even though both viruses were also detected in rodents of other species (Table 3). SWSV and ASIV were found exclusively in common shrews (Table 4). Differences in prevalence rates between female and male hosts were not statistically significant on the level of localities or on the level of the individual host species (for detailed results, see Table S3 in the supplemental material).

**TABLE 3:** Prevalence of TULV and KURV RNAs in rodents and shrews from the Czech Republic^*a*^

Species of tested animal	Prevalence (%) (no. of positive animals/no. of animals tested)		
	TULV	KURV	Total
*Microtus arvalis*	70.8 (17/24)	8.3 (2/24)	79.2 (19/24)
*Clethrionomys glareolus*	10.0 (2/20)	0 (0/20)	10.0 (2/20)
Apodemus agrarius	10.9 (5/46)	15.2 (7/46)	26.1 (12/46)
*Apodemus sylvaticus*	8.0 (2/25)	8.0 (2/25)	16.0 (4/25)
*Apodemus flavicollis*	5.3 (2/38)	0 (0/38)	5.3 (2/38)

a Viral RNA was detected by nested reverse transcription-PCR (RT-PCR) with universal primer pairs targeting orthohantavirus RNA in all available tissue samples. Orthohantaviruses were identified based on the sequencing of a portion of the large (and medium) segment of orthohantavirus genomic RNA. TULV, Tula virus; KURV, Kurkino virus.

**TABLE 4:** Prevalence of SWSV and ASIV RNAs in rodents and shrews from the Czech Republic^*a*^

Species of tested animal	Prevalence (%) (no. of positive animals/no. of animals tested)		
	SWSV	ASIV	Total
*Sorex araneus*	50.0 (2/4)	25.0 (1/4)	75.0 (3/4)
*Sorex minutus*	0 (0/1)	0 (0/1)	0 (0/1)
*Crocidura suaveolens*	0 (0/2)	0 (0/2)	0 (0/2)
*Crocidura leucodon*	0 (0/1)	0 (0/1)	0 (0/1)
*Neomys fodiens*	0 (0/2)	0 (0/2)	0 (0/2)

a Viral RNA was detected by nested RT-PCR with universal primer pairs targeting orthohantavirus RNA in all available tissue samples. Orthohantaviruses were identified based on sequencing of a portion of the large segment of orthohantavirus genomic RNA. SWSV, Seewis virus; ASIV, Asikkala virus.

### Phylogenetic analyses.

The final alignment of L segment sequences yielded a 290-bp-long matrix containing 97 sequences of orthohantaviruses; the final alignment of M segment sequences was 292 bp long and contained 39 sequences of orthohantaviruses. Phylogenetic analyses of both matrices produced well-resolved trees with a basic structure corresponding to the phylogenies presented previously by Klempa et al. (7) and Zelená et al. (35). However, the addition of DOBV, KURV, TULV, SWSV, ASIV, and other orthohantaviruses to the common phylogeny has made the overall evolutionary picture of the genus *Orthohantavirus* even more complex.

All 9 KURV sequences of the L segment obtained from our samples, which originated from striped field mice (6 sequences), common voles (2 sequences), and a yellow-necked mouse (1 sequence), were placed onto the KURV branch. They were split into two distinct clusters regardless of the host species, locality, or tissue type (see Fig. 2). For the M segment, we managed to obtain only a single sequence from samples previously positive for KURV (according to the L segment sequence). That sequence was obtained from a striped field mouse and could not be assigned to a specific virus clade as the whole *Dobrava-Belgrade orthohantavirus* cluster remained unresolved in the M segment tree (see Fig. 3).

We obtained 28 TULV sequences of the L segment, which originated from common voles (18 sequences), striped field mice (5 sequences), bank voles (2 sequences), wood mice (2 sequences), and a yellow-necked mouse (1 sequence). They branched within two phylogenetically distinct clusters based on the sampled localities. One of the branches was associated almost exclusively with samples from Vestec (see Fig. 2). Fewer TULV sequences were obtained for the M segment (18 sequences), but they still indicated the same pattern of two distinct clusters (see Fig. 3).

Two sequences of the L segment from common shrews clustered with SWSV sequences, while one sequence represented ASIV. Unfortunately, we did not manage to sequence the M segment of any samples from shrews despite multiple efforts.

### Tissue tropism.

Concerning the tissue specificity and efficiency of orthohantavirus RNA detection, virus-specific patterns were observed. TULV was most efficiently detected in the lung tissue (82% of the individuals positive in any tissue), whereas KURV was more efficiently detected in the liver (71%), the spleen (71%), and, most surprisingly, the brain (75%) (Table 5). No TULV-positive kidney samples were found in the tested mice or bank voles, including 6 samples from individuals positive in other tissues, whereas the same virus was efficiently detected in the kidney tissues of 65% of the positive common voles (Table S4). Nevertheless, the differences in the prevalences of TULV and DOBV in the individual tissue samples were not statistically significant. Shrew-borne orthohantaviruses were found in the lungs, liver, brain, and heart tissue (Table S4).

**TABLE 5:** Tissue tropism and efficiency of detection of orthohantavirus RNA in different tissue samples from orthohantavirus RNA-positive individuals^*a*^

Virus	No. of positive individuals	% positive samples (no. of positive samples/no. of positive individuals with sample available)					
		Lungs	Kidneys	Liver	Spleen	Brain	Heart
TULV	28	82.1 (23/28)	52.4 (11/21)	65.2 (15/23)	16.7 (1/6)	0 (0/2)	NA
KURV	9	55.6 (5/9)	0 (0/3)	71.4 (5/7)	71.4 (5/7)	75.0 (3/4)	0 (0/2)
SWSV	2	50.0 (1/2)	0 (0/1)	50.0 (1/2)	0 (0/1)	50.0 (1/2)	100 (1/1)
ASIV	1	100 (1/1)	NA	NA	NA	100 (1/1)	100 (1/1)

Total	40	75.0 (30/40)	44.0 (11/25)	65.6 (21/32)	42.9 (6/14)	55.6 (5/9)	50.0 (2/4)

a The percentage was calculated as the ratio of the number of positive samples of the particular tissue type to the total number of positive individuals with this tissue sample available (not all tissues were sampled from all individuals). TULV, Tula virus; KURV, Kurkino virus; SWSV, Seewis virus; ASIV, Asikkala virus; NA, not available.

## DISCUSSION

Orthohantaviruses are emerging zoonotic pathogens that have a significant impact on human health in many countries (41). Although a similar or even higher seroprevalence has been found in the human population in the Czech Republic, the incidence rate of human cases of orthohantavirus infection is significantly lower than those in other countries in central Europe, especially the neighboring countries Austria, Germany, and Slovakia (42). This could be due to an underestimation of the number of clinical cases, a higher occurrence of clinically inapparent cases, or (most likely) a combination of both. KURV and TULV are among the most frequently detected orthohantaviruses in rodents in the Czech Republic, in both in our study (Table 3) and previous studies (29, 35). Both pathogens are associated with a mild course of the disease (43, 44). In contrast, PUUV has been reported to be a major cause of human infection elsewhere in Europe (45) and also in Austria (46) and Germany (43), including areas bordering the Czech Republic. DOBV and KURV human HFRS cases are significantly less frequent in central Europe (43, 47). In the Czech Republic, PUUV, DOBV, and KURV are the most frequent causes of clinically apparent, diagnosed orthohantavirus disease cases in humans (16, 35, 48, 49), although they remain relatively rare and spatially and geographically isolated.

**FIG 1:**
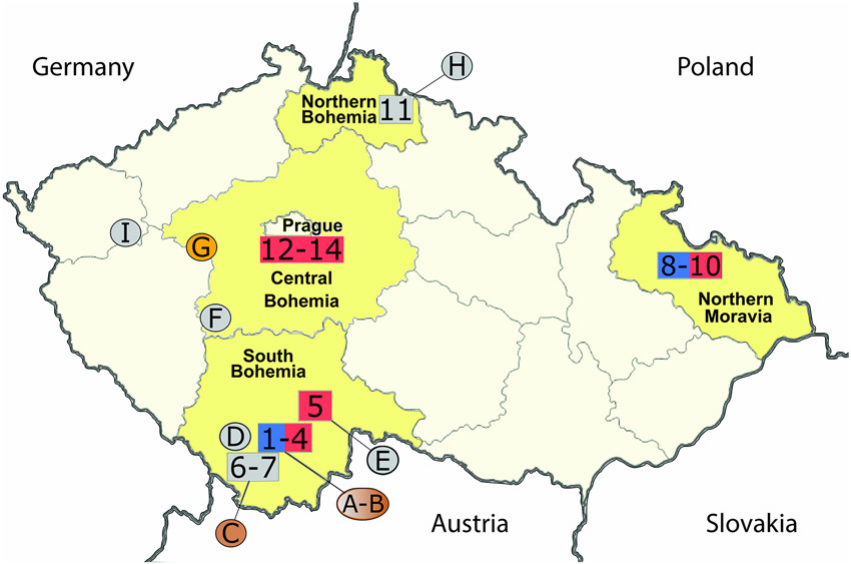
Geographic distribution of the localities used for rodent trapping and places where the dead shrews were found. Localities of rodent trapping are marked by numbers according to Table 6. Localities of the collected shrews are marked by letters according to Table 2. Colors indicate the orthohantaviruses detected (red, Tula virus; blue, Kurkino virus; brown, Seewis virus; orange, Asikkala virus; gray, locality where no orthohantavirus RNA-positive samples were detected). (The map template is from https://commons.wikimedia.org/wiki/File:Czechia_-_colored_blank_map.png.)

KURV was detected mainly in striped field mice, two wood mice, and two common voles (Table 3). The presence of the related DOBV was previously reported in 2 yellow-necked mice in Northern Moravia (35) and in rodents of multiple species in South Bohemia (50). Interestingly, in our study, KURV was detected in multiple individuals at the two trapping sites in Northern Moravia and one trapping site in South Bohemia (Fig. 1). The nucleotide sequences obtained from both regions clustered with sequences from rodents and human patients from Northern Moravia (35). The authors of that previous study (35) mentioned that DOBV was detected more frequently in mountainous areas, whereas KURV was associated with lowlands; our samples originated from lowlands.

In our study, PUUV was not detected in any of the 20 bank voles or animals of any other species. There is a single study reporting the direct detection of PUUV in rodents in the Czech Republic (49), indicating that the distribution of this virus might be highly focal. As also previously reported (29, 51, 52), TULV is prevalent among populations of common voles in the Czech Republic. Although it is rarely detected in humans, infections of immunocompromised (33) as well as immunocompetent (32, 47, 53) patients were reported. In general, the distribution of orthohantaviruses in their reservoir hosts, as well as the distribution of human cases, is influenced by numerous factors on the side of the reservoirs, the virus, and the human population (42, 54), resulting in high spatiotemporal variability (43).

Phylogenetic analyses of the L segment indicate that the detected TULV and shrew-borne orthohantaviruses are strictly monophyletic. The members of the *Dobrava-Belgrade orthohantavirus* species split into 4 monophyletic lineages according to the individual viruses, DOBV, KURV, SAAV, and SOCV, which is in congruence with data from previous publications by Klempa et al. (7) and Zelená et al. (35). Our sequences were classified as KURV. Similarly, it seems obvious that TULV is not composed of a single genotype, but it also splits into several distinct genotypes within central Europe, regardless of the reservoir host (43, 55, 56). Since little is known of its pathogenicity to humans, we cannot assess whether this differentiation may have any significance in terms of the impact on human health (i.e., that one lineage may be more pathogenic than the other). Data from phylogenetic analyses of the M segment were congruent with the results of Klempa et al. (7), suggesting that the phylogenetic position of SAAV is unresolved, being scattered among the viruses of the *Dobrava-Belgrade orthohantavirus* species. The phylogram of the M segment was less resolved than that of the L segment. The M segment, encoding the Gn and Gc surface glycoprotein precursors, is known to undergo faster evolution than the L (RNA-dependent RNA polymerase) and S (nucleocapsid) segments (57, 58), which is reflected in the long branch of TULV in the M segment compared to the L segment phylogenetic tree.

**FIG 2:**
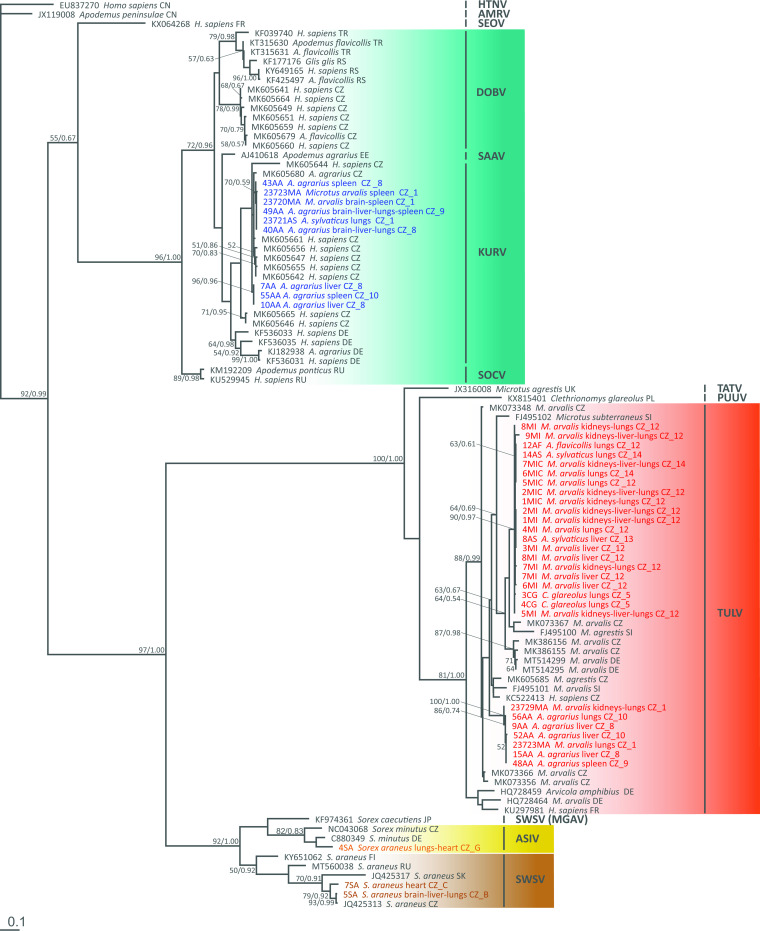
Phylogenetic relationships of the obtained sequences of orthohantaviruses inferred by maximum likelihood (ML) analysis of the RNA-dependent RNA polymerase gene (L segment). The Bayesian inference (BI) tree was mapped onto the ML tree. Numbers at the nodes show bootstrap values derived from the ML analysis/posterior probabilities under the BI analysis. Bootstrap supports and posterior probabilities of <50% and <0.50, respectively, are not provided. Hantaan virus was used as an outgroup. Colors indicate the orthohantaviruses (blue, viruses of the *Dobrava-Belgrade orthohantavirus* species; red, Tula virus; brown, Seewis virus; yellow, Asikkala virus). Accession numbers for the sequences obtained from GenBank are indicated. Each original sample code consists of the abbreviation of the specific code of the sample, the host species, the country code, and the map reference (Fig. 1 and Table 6). HTNV, Hantaan virus; AMRV, Amur virus; SEOV, Seoul virus; DOBV, Dobrava virus; SAAV, Saaremaa virus; KURV, Kurkino virus; SOCV, Sochi virus; TATV, Tatenale virus; PUUV, Puumala virus; TULV, Tula virus; SWSV, Seewis virus; MGAV, Amga virus; ASIV, Asikkala virus; CZ, Czech Republic; DE, Germany; EE, Estonia; FI, Finland; FR, France; JP, Japan; PL, Poland; CN, China; RS, Serbia; RU, Russia; SI, Slovenia; SK, Slovakia; TR, Turkey; UK, United Kingdom.

Orthohantaviruses are considered to be highly host specific (8, 59). In our study, the majority of TULVs were detected in common voles (family Cricetidae), which are typical hosts of the virus in central Europe (29, 30). Similarly, as expected, KURV was most frequently found in striped field mice (Muridae) (7), and SWSV and ASIV were detected exclusively in common shrews (18, 34). Nevertheless, TULV RNA was detected in four striped field mice, two wood mice, two yellow-necked mice, and two bank voles, and likewise, two wood mice and one common vole were positive for KURV RNA. Most of the atypical hosts shared a locality (i.e., lived syntopically) with the positive individuals of the typical host species, and sequence analysis confirmed the high identity of sequences obtained from typical and atypical hosts, indicating interspecies (interfamily) spillover. The possibility of cross-contamination can never be eliminated, but we have taken measures to minimize this risk. In addition, the virus was detected in multiple tissues from the same individual infected with an atypical orthohantavirus, and the individuals originated from different trapping sites and trapping events, which makes accidental cross-contamination highly unlikely. The possibility of infection of bank voles with TULV as well as infection of mice (yellow-necked mice and laboratory mice) with atypical viruses of the *Dobrava-Belgrade orthohantavirus* species was partially confirmed in a previous laboratory experiment (60). There is evidence that spillover infection occurs under natural conditions between host species belonging to the same family (50, 56, 61, 62) rather than between members of different families (35, 50). However, the exclusive use of the typical host even under conditions of sympatric/syntopic occurrence of the hosts and viruses has also been reported (4, 63). On the other hand, surveillance of hantaviruses often focuses on a particular host species and/or a particular virus; therefore, the frequency of intergenus spillover may be underestimated. Our data do not allow us to assess whether infection of an atypical host results in the same course of infection and whether and how effectively atypical hosts may participate in virus circulation in nature. Nevertheless, our records of KURV and TULV hantavirus spillover to hosts of different families indicate possible lower host specificity and the potential for hantavirus coinfections. Interestingly, one striped field mouse (52AA) (only a short KURV sequence was available, which was not included in the phylogenetic analysis) and one common vole (23723MA) were found to be infected simultaneously by KURV and TULV (Fig. 2). Although each of the viruses was detected in a different organ, such coinfection can lead to reassortment or recombination events (39) because the two viruses may encounter each other in the same tissue at a different stage of infection.

**FIG 3:**
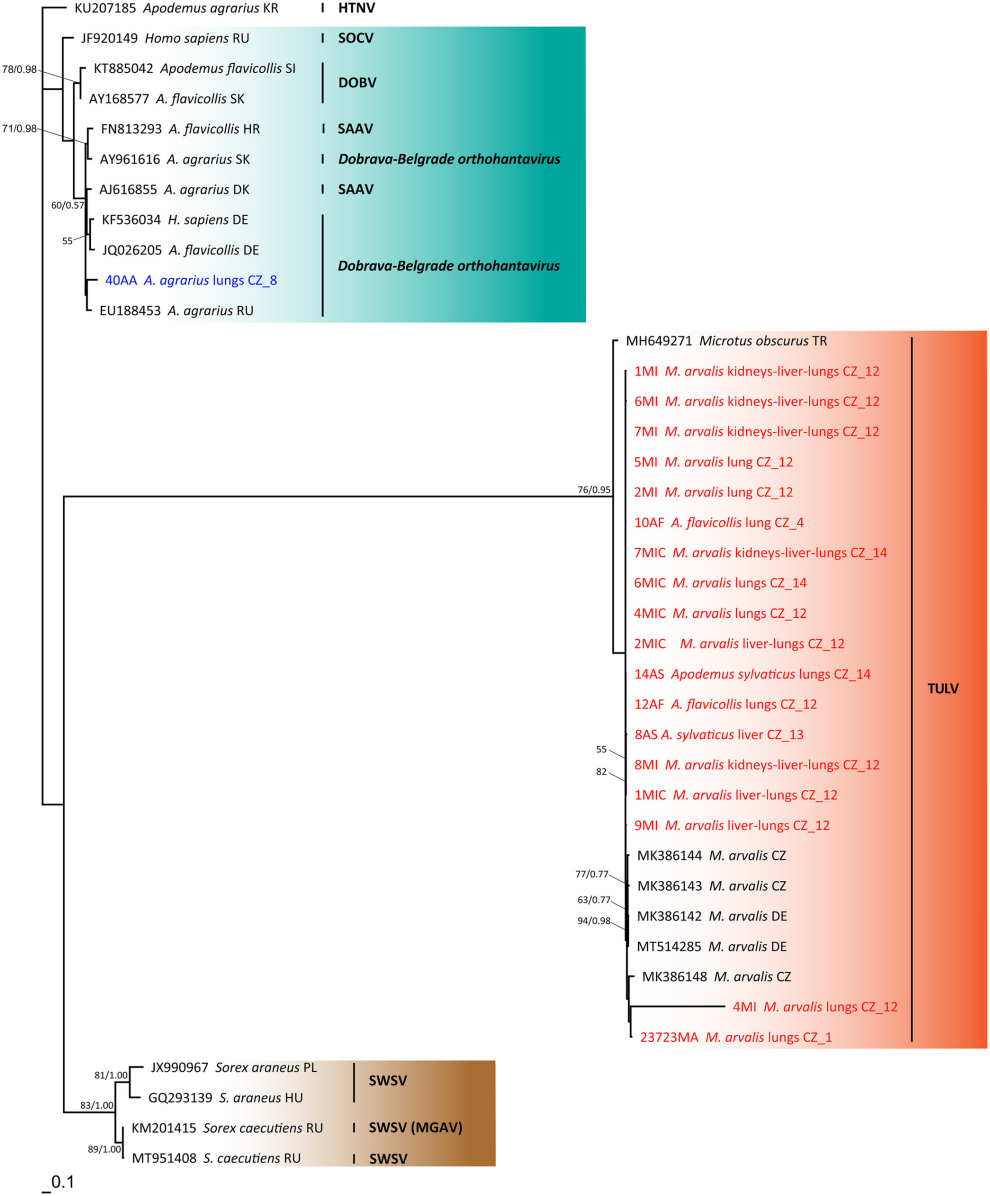
Phylogenetic relationships of the obtained sequences of orthohantaviruses inferred by maximum likelihood (ML) analysis of the glycoprotein precursor gene (M segment). The Bayesian inference (BI) tree was mapped onto the ML tree. Numbers at the nodes show bootstrap values derived from the ML analysis/posterior probabilities under the BI analysis. Bootstrap supports and posterior probabilities of <50% and <0.50, respectively, are not provided. Hantaan virus was used as an outgroup. Colors indicate the orthohantaviruses (blue, viruses of the *Dobrava-Belgrade orthohantavirus* species; red, Tula virus; brown, Seewis virus). Accession numbers for the sequences obtained from GenBank are indicated. Each original sample code consists of the abbreviation of the specific code of the sample, the species of the host, the country code, and the map reference (Fig. 1 and Table 6). HTNV, Hantaan virus; SOCV, Sochi virus; DOBV, Dobrava virus; SAAV, Saaremaa virus; TULV, Tula virus; SWSV, Seewis virus; CZ, Czech Republic; DE, Germany; HR, Croatia; HU, Hungary; KR, South Korea; PL, Poland; SI, Slovenia; SK, Slovakia; RU, Russia; TR, Turkey.

Orthohantaviruses, as viruses with a segmented genome, may exchange segments and form reassortants. Unlike orthobunyaviruses, they usually form reassortants within members of the same virus or virus species rather than between two different virus species. The M segment is most likely to be replaced, while the combination of L and S segments usually remains stable (39). Evidence of reassortments is usually revealed as a conflicting topology of virus nucleotide sequences of each genomic segment from the same host individual. Therefore, we compared the phylogenetic position of the L segment sequences to their position in the M segment phylogenetic tree (Fig. 2 and 3). No evidence of interspecies reassortment was found. Nevertheless, while one TULV sequence obtained from a common vole trapped in the Praha-západ district (4MI) grouped with all other sequences from the same locality in the L segment-based phylogenetic tree (Fig. 1), its position in the M segment-based phylogeny indicates possible reassortment between two TULV lineages (Fig. 2). However, because only short sequences of both genome fragments were available, we are not able to distinguish between reassortment and homologous recombination (39).

Most studies on trapped rodents have screened only a single tissue type, usually the lungs (21, 35, 49) or the kidneys (63), for orthohantavirus detection. Because there may be differences in the efficiencies of orthohantavirus detection in different tissues, we compared the rates of detection of TULV and KURV in positive individuals in all different available tissue samples. Although the differences were not statistically significant (possibly because of the insufficient number of positive samples and incomplete tissue sample sets from several individuals [see Table S1 in the supplemental material]), our results generally confirmed the observations from previous studies, namely, the low efficiency of detection of KURV (DOBV) compared with TULV in the lungs, the high efficiency of orthohantavirus detection in the liver, and the possibility of detecting orthohantaviral RNA in brain tissues of rodents and shrews (Table S4) (15, 56, 64, 65). Based on our results, we hypothesize that the tissue tropism is virus specific not only in humans but also in natural orthohantavirus rodent hosts and that infection is often multisystemic. These observations need to be confirmed on a larger scale and with a complete sample set that would allow adequate statistical evaluation. Nevertheless, our pilot findings are of great importance because these mechanisms may significantly affect the overall efficiency of orthohantaviral RNA detection.

In addition to rodent-associated orthohantaviruses, RNAs of the shrew-borne orthohantaviruses SWSV and ASIV were also detected in our study. Considering the fact that the shrews were found completely randomly at different, geographically distant locations and yet 3 out of 10 were positive for orthohantavirus RNA (only common shrews), we assume that there is a high prevalence of these orthohantaviruses in shrews in the Czech Republic. SWSV has already been detected several times in central Europe (34, 66), particularly in the Czech Republic (31, 35). Our L segment sequences obtained from common shrews formed a well-supported separate intracluster within the SWSV clade. It is evident that all three sequences from the Czech Republic are distinct from those from Slovakia, Russia, and Finland (19, 67). The SWSV L segment sequence in the GenBank database under accession number JQ425313 (19), from a common shrew, originates from the same district, České Budějovice, where we detected the SWSV-positive sample 5SA. Concerning the time gap between the detection of the two positive common shrews (11 years) and the 99% L segment nucleotide identity (328/330), we can state that after all of these years, SWSV in České Budějovice is still present and circulates in shrews in this area almost unchanged. We also detected ASIV in another common shrew (sample 4SA). ASIV was detected in the Czech Republic and neighboring Germany in both common shrews and Eurasian pygmy shrews. The sympatric occurrence of these species provides an opportunity for spillover infections; however, phylogenetic analyses and the broad geographic distribution of ASIV across Europe in Eurasian pygmy shrews imply that shrews of this species are the primary reservoir hosts (18).

In conclusion, we detected multiple orthohantaviruses in free-living rodents and shrews in the Czech Republic. Moreover, our data suggest possible virus-specific tissue tropism in rodent hosts, a high prevalence of SWSV in common shrews, and a high prevalence of TULV in common voles (with frequent spillover to hosts of other species, including Muridae) in the Czech Republic. Since most of the rodents were trapped in the vicinity of human settlements, and human-pathogenic KURV and potentially pathogenic TULV were found, our results suggest a potential risk to public health.

## MATERIALS AND METHODS

### Ethical statements.

This study included the trapping of free-living rodents. The trapping and manipulation of the trapped animals were carried out in strict accordance with Czech national laws and guidelines on the use of experimental animals and protection of animals against cruelty (Animal Welfare Act number 246/1992 Coll.). The protocol was approved by the Committee on the Ethics of Animal Experiments of the University of South Bohemia and by the Ministry of the Environment of the Czech Republic (permit numbers 51304/ENV/14-2981/630/14, MZP/2017/630/854, and MZP/2021/630/2459).

### Sampling.

From 2016 to 2021, rodents (yellow-necked field mice, striped field mice, wood mice, common voles, and bank voles) were live trapped in 14 areas of the Czech Republic (Table 6 and Fig. 1). Furthermore, randomly found cadavers of shrews (10 individuals) were collected and also subjected to the screening process (Table 2 and Fig. 1).

**TABLE 6:** Detailed information on the localities of rodent trapping

Locality ID	Locality; district (region)	Locality type	GPS coordinates (WGS84)	Yr(s) of collection
1	Borek; České Budějovice (South Bohemia)	Urban area	49°00′45.677″N, 14°29′46.141″E	2016
2	Vltava; České Budějovice (South Bohemia)	Urban area (housing estate)	48°59′56.238″N, 14°27′19.339″E	2017
3	Mánesova Street no. 273/9; České Budějovice (South Bohemia)	Urban area (house cellar)	48°58′09.730″N, 14°28′45.020″E	2018
4	Švábův Hrádek; České Budějovice (South Bohemia)	Rural area (weed)	48°58′16.600″N, 14°26′20.212″E	2020
5	Lužnice, field station U Zahradníků no. 92; Jindřichův Hradec (South Bohemia)	Rural area (congress center)	49°04′51.428″N, 14°45′41.266″E	2018
6	Zbytiny-Koryto; Prachatice (South Bohemia)	Area of confirmed hantavirus disease in humans	48°55′53.899″N, 14°01′23.761″E	2018
7	Květušín; Český Krumlov (South Bohemia)	Area of confirmed hantavirus disease in humans	48°46′56.620″N, 14°07′59.710″E	2021
8	Oldřišov; Opava (Northern Moravia)	Rural area (agricultural)	49°58′36.249″N, 17°57′30.491″E	2016
9	Oldřišov, sugar beet field between Oldřišov and Opava; Opava (Northern Moravia)	Rural area (agricultural)	49°59′04.414″N, 17°56′47.773″E	2016
10	Weed hill near Hillova Street; Opava (Northern Moravia)	Urban area	49°57′11.994″N, 17°54′55.937″E	2016
11	Varnsdorf; Děčín (Northern Bohemia)	Rural area (agricultural)	50°55′09.899″N, 14°35′53.808″E	2018, 2019
12	Vestec, Biocev; Praha-západ (Central Bohemia)	Urban area (research center complex)	49°58′54.020″N, 14°29′16.572″E	2020
13	Vestec, near the Shell gas station; Praha-západ (Central Bohemia)	Urban area	49°59′34.318″N, 14°29′32.185″E	2020
14	Dolní Břežany; Praha-západ (Central Bohemia)	Urban area	49°57′44.389″N, 14°27′57.209″E	2020

Sherman live traps (LFA size; H. B. Sherman Traps, Inc., Tallahassee, FL, USA) filled with bait were set in the late evening, spaced approximately 10 m apart, and left in the field overnight. The lungs and occasionally also other visceral organs, liver, kidneys, spleen, brain, and heart, were sampled directly after the animal was killed by cervical dislocation and preserved in an RNA stabilization solution (RNAlater; Invitrogen, Vilnius, Lithuania). Sterile dissection tools were used for each individual and cleansed between samplings of the individual organs. After transport to the laboratory, the samples were stored at −80°C. Detailed data on individual rodents are presented in Table S1 in the supplemental material.

Reservoir hosts of species with overlapping morphologies that are difficult to be distinguished in the field (yellow-necked field mice, wood mice, and shrews) were identified by methods of molecular biology (diagnostic PCR and sequencing) (68, 69).

### RNA extraction and reverse transcription.

Individual rodent tissue samples were cleansed from RNAlater and homogenized in sterile phosphate-buffered saline (PBS) as 10% (wt/vol) (liver) or 20% (wt/vol) (all remaining tissue samples) suspensions using an automated homogenizer (Tissue Lyzer II; Qiagen, Hilden, Germany) and sterile 5-mm stainless steel beads at 30 Hz for 2 min (Qiagen, Hilden, Germany). After centrifugation, the supernatant was collected, and RNA isolation was performed using a commercially available silica column-based kit (QIAamp viral RNA minikit; Qiagen, Hilden, Germany) according to the manufacturer’s instructions. Using a high-capacity RNA-to-cDNA kit (Thermo Fisher Scientific, Vilnius, Lithuania) and 5 μL of total RNA as the template, cDNA was synthesized according to the manufacturer’s instructions.

### PCR amplification and sequencing. (i) Screening PCR.

All of the available samples were screened for orthohantavirus RNA. Nested PCR with primer pairs Han-L-F1 and Han-L-R1 (first reaction) and Han-L-F2 and Han-L-R2 (second reaction) (Table 7) was used to amplify the partial sequences of the orthohantaviral L segment encoding the RNA-dependent RNA polymerase (70). The first PCR was carried out using a total volume of 25 μL, including 1.0 μL of each primer (10 μM), 12.5 μL of PCR master mix (Combi PPP master mix; Top-Bio, s. r. o., Vestec, Czech Republic), 6.5 μL of PCR-grade water, and 4 μL of synthesized cDNA. The annealing temperature was set based on the best result of the gradient PCR. Parameters for nested PCRs were as follows: an initial denaturation step at 95°C for 6 min, followed by 40 cycles of denaturation at 95°C for 30 s, annealing at 53°C for 30 s, and extension at 72°C for 30 s. The final extension step was performed at 72°C for 3 min. Subsequently, 1 μL of the product of the first PCR was used for the nested reaction according to the same protocol (the missing volume in the PCR mix was filled with PCR-grade water). Individual steps of the detection protocol (nucleic acid extraction, preparation of PCR master mixes, amplification, electrophoresis, and PCR product purification) were performed in separate rooms, using separate equipment. Moreover, PCR master mixes were prepared in a dedicated PCR box, samples and isolated nucleic acids were handled in biohazard boxes, and all working surfaces were decontaminated using bleach and UV light before and after the work.

**TABLE 7:** Primers used for the screening of rodent tissue samples and sequencing of orthohantavirus-positive samples

Primer name	Primer sequence	Direction^*a*^	Annealing temp (°C)	Approximate size of PCR product (bp)	Target	Reference
HAN-L-F1	ATGTAYGTBAGTGCWGATGC	F	53	420	L segment	70
HAN-L-R1	AACCADTCWGTYCCRTCATC	R				
HAN-L-F2	TGCWGATGCHACIAARTGGTC	F	53	390		
HAN-L-R2	GCRTCRTCWGARTGRTGDGCAA	R				
1470c	CCIGGITTICATGGITGGGC	F	40	600	DOBV M segment	16
2029R	CCATGIGCITTITCIKTCCA	R				
1674F	TGTGAIKTITGIAAITAIGAGTGTGA	F	40	320		
1990R	TCIGMTGCISTIGCIGCCCA	R				
28F	AATTGAAAAGGTGAAGCAGG	F	50	460	TULV M segment	This study
492R	GCAGATGATGGTAGGGAAAA	R				

a F, forward; R, reverse.

### (ii) M segment-specific PCR.

Samples positive for RNA of the viruses belonging to the *Dobrava-Belgrade orthohantavirus* species (according to the sequencing of the screening PCR product) were submitted for amplification of the partial sequence of the orthohantaviral M segment encoding the Gn and Gc glycoprotein precursors. The PCR mixtures were prepared as described above for the screening nested PCR, employing the primer pairs 1470c and 2029R (first PCR) and 1674F and 1990R (second PCR) (16) (Table 7). The parameters for PCR were as follows: an initial denaturation step at 95°C for 6 min, followed by 40 cycles of denaturation at 95°C for 30 s, annealing at 40°C for 30 s, and extension at 72°C for 30 s. The final extension step was performed at 72°C for 3 min. Primer pair 28F and 492R (Table 7) was used for TULV-positive samples, according to the protocol and parameters described above, except that the annealing temperature was 50°C.

### Processing of the PCR products and sequencing.

PCR amplicons were visualized on a 2% agarose gel using Sybr green (Life Technologies Europe, Bleiswijk, the Netherlands) under UV light (Uvitec, Cambridge, United Kingdom). PCR products of the expected sizes were purified using 0.2 μL of FastAP (thermosensitive alkaline phosphatase) and 0.2 μL of Exo I (exonuclease I from Escherichia coli) enzymes (Thermo Fisher Scientific, Waltham, MA, USA). Enzymatic digestion was carried out in a thermocycler at 37°C for 15 min, followed by enzyme inactivation at 80°C for 15 min. The purified PCR products were directly sequenced via the Sanger sequencing method by Macrogen, Inc. (Amsterdam, the Netherlands), on an automatic 3730XL DNA analyzer (https://www.macrogen-europe.com/services/sanger-sequencing/standard). The obtained sequences were verified by the BLAST algorithm (https://blast.ncbi.nlm.nih.gov/Blast.cgi) and adjusted using Sequence Scanner v2.0 (https://products.appliedbiosystems.com). The EditSeq and SeqMan v5.05 programs (DNASTAR Inc., Madison, WI, USA) were used to assemble the sequences.

### Phylogenetic analyses.

The obtained partial sequences of the L and M genomic segments of orthohantaviruses from rodents and shrews, together with the sequences of related orthohantaviruses available in the GenBank database, were used for phylogenetic analyses. The data set was aligned with the BioEdit v7.2.5 program (71), using the ClustalW multiple-alignment algorithm (72). The resultant alignment was manually trimmed to a uniform length. For the reconstruction of phylogenetic relationships, two approaches were used: Bayesian inference (BI), performed using MrBayes v3.2.2 (73), and maximum likelihood (ML), performed using PhyML v2.4.3 (74). The most suitable evolutionary models were selected by jModeltest (75, 76). BI analysis was calculated under the GTR+Γ+I evolutionary model; the Markov chain Monte Carlo (MCMC) method was specified for 10 million generations, with a frequency of collection of every 500 generations, and the burn-in was set to 25%. ML was also conducted using the GTR+Γ+I model, and bootstrap values were calculated with 1,000 replicates. The resultant phylogenetic trees were visualized and exported in TreeView v1.6.6 (77) and graphically edited in Adobe Illustrator CC v2017.0.2 (Adobe Systems, Inc.).

### Statistical analyses.

Differences in orthohantavirus prevalences between female and male hosts as well as differences in the prevalences of particular orthohantavirus species in individual tissue samples were tested using Fisher’s exact test (GraphPad Prism v9.3.1; GraphPad Software, CA, USA). Differences with *P* values of <0.05 were considered statistically significant.

### Data availability.

Nucleotide sequences were deposited in the NCBI GenBank database (www.ncbi.nlm.nih.gov) under the accession numbers ON243777 to ON243817 and ON653425 to ON653442 (see Tables S2 and S3 in the supplemental material).
