# Supplementary material for: Orthohantaviruses in Reservoir and Atypical Hosts in the Czech Republic: Spillover Infection and Indication of Virus-Specific Tissue Tropism
Source: Microbiol Spectr. 2022 Sep 28;10(5):e01306-22. doi: 10.1128/spectrum.01306-22 (PMC9604079; doi:10.1128/spectrum.01306-22)
Supplement: Supplemental file 1 — Tables S1 to S4. Download spectrum.01306-22-s0001.pdf, PDF file, 0.5 MB [file spectrum.01306-22-s0001.pdf]

Supplementary Table S1. Detailed summary of all screened samples for hantavirus RNA in this study. AF stands for *Apodemus flavicollis*, AS – *A. sylvaticus*, AA – *A. agrarius*, MA – *Microtus arvalis*, CG – *Clethrionomys glareolus*, SM – *Sorex minutus,* SA – *Sorex araneus,* CL – *Crocidura leucodon,* CS – *Crocidura suaveolens,* NF – *Neomys fodiens.*

Sample code	Locality	District	Year of collection	Host speci es	Sex	Age	Sampled Tissue	Virus detected, positive tissue
23719AS_BO	Borek	České Budějovice	2016	AS	M	SUB	Lungs, kidneys	-
23720MA_BO	Borek	České Budějovice	2016	MA	M	SUB	LungsLungss, liver, kidneys, spleen, brain, heart	KURV<br>liver, spleen, brain
23721AS_BO	Borek	České Budějovice	2016	AS	F	SUB	Lungs, kidneyskidneyss, brain	KURV<br>lungs
23722AF_BO	Borek	České Budějovice	2016	AF	F	SUB	Lungs, liver, kidneys, brain, heart	-
23723MA_BO	Borek	České Budějovice	2016	MA	M	SUB	Lungs, kidneys, spleen	TULV<br>lungs<br>KURV<br>spleen
23725AS_BO	Borek	České Budějovice	2016	AS	F	AD	Lungs, kidneys	-
23727AS_BO	Borek	České Budějovice	2016	AS	M	AD	Lungs, kidneys, heart	KURV<br>lungs
23728MA_BO	Borek	České Budějovice	2016	MA	F	SUB	Lungs, kidneys	-
23729MA_BO	Borek	České Budějovice	2016	MA	F	SUB	Lungs, kidneys	TULV<br>lungs, kidneys
23920AS_VLT	Vltava	České Budějovice	2017	AS	-	-	Lungs, liver, spleen, brain	-
23921AF_VLT	Vltava	České Budějovice	2017	AF	-	-	Lungs, liver, kidneys, brain	-

23922AS_VLT	Vltava	České Budějovice	2017	AS	-	-	Lungs, liver, kidneys, brain	-
23923AF_VLT	Vltava	České Budějovice	2017	AF	-	-	Lungs, liver, kidneys, brain	-
23924AF_VLT	Vltava	České Budějovice	2017	AF	-	-	Lungs, liver, kidneys, brain	-
23925AF_VLT	Vltava	České Budějovice	2017	AF	-	-	Lungs, liver, kidneys, brain	-
23926AF_VLT	Vltava	České Budějovice	2017	AF	-	-	Lungs, liver, kidneys, brain	-
23927AS_VLT	Vltava	České Budějovice	2017	AS	-	-	Lungs, liver, kidneys, brain	-
23928CG_VLT	Vltava	České Budějovice	2017	CG	-	-	Lungs, liver, kidneys, brain	-
23929AF_VLT	Vltava	České Budějovice	2017	AF	-	-	Lungs, liver, kidneys, brain	-
23930AF_VLT	Vltava	České Budějovice	2017	AF	-	-	Lungs, liver, kidneys, brain	-
23931CG_VLT	Vltava	České Budějovice	2017	CG	-	-	Lungs, liver, kidneys, brain	-
23932AF_VLT	Vltava	České Budějovice	2017	AF	-	-	Lungs, liver, kidneys, brain	-
23933AS_VLT	Vltava	České Budějovice	2017	AS	-	-	Lungs, liver, kidneys, brain	-
23935CG_VLT	Vltava	České Budějovice	2017	CG	-	-	Lungs, liver, kidneys, brain	-
23936CG_VLT	Vltava	České Budějovice	2017	CG	-	-	Lungs, liver, kidneys, brain	-
23937AF_VLT	Vltava	České Budějovice	2017	AF	-	-	Lungs, liver, kidneys, brain	-

23938CG_VLT	Vltava	České Budějovice	2017	CG	-	-	Lungs, liver, kidneys, brain	-
23940CG_VLT	Vltava	České Budějovice	2017	CG	-	-	Lungs, liver, kidneys, brain	-
23941CG_VLT	Vltava	České Budějovice	2017	CG	-	-	Lungs, liver, kidneys, brain	-
1AF_MAN	České Budějovice, Mánesova street no.273/9	České Budějovice	2018	AF	-	-	Lungs, liver, kidneys, spleen, brain, heart	-
2AS_MAN	České Budějovice, Mánesova street no.273/9	České Budějovice	2018	AS	M	-	Lungs, liver, kidneys, spleen, brain, heart	-
3AS_MAN	České Budějovice, Mánesova street no.273/9	České Budějovice	2018	AS	F	-	Lungs, liver, kidneys, spleen, brain, heart	-
4AS_MAN	České Budějovice, Mánesova street no.273/9	České Budějovice	2018	AS	M	-	Lungs, liver, kidneys, spleen, brain, heart	-
5AS_MAN	České Budějovice, Mánesova street no.273/9	České Budějovice	2018	AS	M	-	Lungs, liver, kidneys, spleen, brain, heart	-
6AF_MAN	České Budějovice, Mánesova street no.273/9	České Budějovice	2018	AF	M	-	Lungs, liver, kidneys, spleen, brain, heart	-
7AF_MAN	České Budějovice, Mánesova street no.273/9	České Budějovice	2018	AF	-	-	Lungs, liver, kidneys, spleen, brain, heart	-

8AF_MAN	České Budějovice, Mánesova street no.273/9	České Budějovice	2018	AF	-	-	Lungs, liver, kidneys, spleen, brain, heart	-
10AF_VES	Švábův Hrádek	České Budějovice	2020	AF	M	AD	Lungs, liver, kidneys	TULV lungs
1AF_LUŽ	Lužnice, field station U Zahradníků no. 92	Jindřichův Hradec	2018	AF	-	-	Lungs	-
3CG_LUŽ	Lužnice, field station U Zahradníků no. 92	Jindřichův Hradec	2018	CG	-	-	Lungs	TULV lungs
4CG_LUŽ	Lužnice, field station U Zahradníků no. 92	Jindřichův Hradec	2018	CG	-	-	Lungs	TULV lungs
5CG_LUŽ	Lužnice, field station U Zahradníků no. 92	Jindřichův Hradec	2018	CG	-	-	Lungs	-
6CG_LUŽ	Lužnice, field station U Zahradníků no. 92	Jindřichův Hradec	2018	CG	-	-	Lungs	-
8CG_LUŽ	Lužnice, field station U Zahradníků no. 92	Jindřichův Hradec	2018	CG	-	-	Lungs	-
10CG_LUŽ	Lužnice, field station U Zahradníků no. 92	Jindřichův Hradec	2018	CG	-	-	Lungs	-
11CG_LUŽ	Lužnice, field station U Zahradníků no. 92	Jindřichův Hradec	2018	CG	-	-	Lungs	-
12CG_LUŽ	Lužnice, field station U Zahradníků no. 92	Jindřichův Hradec	2018	CG	-	-	Lungs	-
13CG_LUŽ	Lužnice, field station U Zahradníků no. 92	Jindřichův Hradec	2018	CG	-	-	Lungs	-
14CG_LUŽ	Lužnice, field station U Zahradníků no. 92	Jindřichův Hradec	2018	CG	-	-	Lungs	-
1AS_ZBY	Zbytiny – Koryto	Prachatice	2018	AS	F	JUV	Lungs, liver, kidneys, spleen, brain, heart	-

2AS_ZBY	Zbytiny – Koryto	Prachatice	2018	AS	-	AD	Lungs, liver, kidneys, spleen, brain, heart	-
3AS_ZBY	Zbytiny – Koryto	Prachatice	2018	AS	F	AD	Lungs, liver, kidneys, spleen, brain, heart	-
4AS_ZBY	Zbytiny – Koryto	Prachatice	2018	AS	F	AD	Lungs, liver, kidneys, spleen, brain, heart	-
5AS_ZBY	Zbytiny – Koryto	Prachatice	2018	AS	F	AD	Lungs, liver, kidneys, spleen, brain, heart	-
6CG_ZBY	Zbytiny – Koryto	Prachatice	2018	CG	M	AD	Lungs, liver, kidneys, spleen, brain, heart	-
8CG_ZBY	Zbytiny – Koryto	Prachatice	2018	CG	F	AD	Lungs, liver, kidneys, spleen, brain, heart	-
1AS_KV	Květušín	Český Krumlov	2021	AS	F	-	Lungs, liver, kidneys, spleen, brain, heart	-
2AF_KV	Květušín	Český Krumlov	2021	AF	F	-	Lungs, liver, kidneys, spleen, brain, heart	-
3AF_KV	Květušín	Český Krumlov	2021	AF	F	-	Lungs, liver, kidneys, spleen, brain, heart	-
4MA_KV	Květušín	Český Krumlov	2021	MA	F	-	Lungs, liver, kidneys, spleen, brain, heart	-
5MA_KV	Květušín	Český Krumlov	2021	MA	M	-	Lungs, liver, kidneys, spleen, brain, heart	-
1AF_O	Oldřišov	Opava	2016	AF	F	AD	Lungs, liver, spleen	-
2AA_O	Oldřišov	Opava	2016	AA	-	AD	Lungs, liver, spleen	-
3AS_O	Oldřišov	Opava	2016	AS	-	JUV	Lungs, liver, spleen	-
7AA_O	Oldřišov	Opava	2016	AA	F	AD	Lungs, liver, spleen	KURV liver

8AF_O	Oldřišov	Opava	2016	AF	M	AD	Lungs, liver, spleen	-
9AA_O	Oldřišov	Opava	2016	AA	F	AD	Lungs, liver, spleen	TULV<br>liver
10AA_O	Oldřišov	Opava	2016	AA	F	SUB	Lungs, liver, spleen	KURV<br>liver
11AA_O	Oldřišov	Opava	2016	AA	F	JUV	Lungs, liver, spleen	-
12AA_O	Oldřišov	Opava	2016	AA	F	SUB	Lungs, liver, spleen	-
13AF_O	Oldřišov	Opava	2016	AF	F	AD	Lungs, liver, spleen	-
14AA_O	Oldřišov	Opava	2016	AA	M	SUB	Lungs, liver, spleen	-
15AA_O	Oldřišov	Opava	2016	AA	F	SUB	Lungs, liver, spleen	TULV<br>liver
16AA_O	Oldřišov	Opava	2016	AA	M	AD	Lungs, liver	-
17AA_O	Oldřišov	Opava	2016	AA	F	AD	Lungs, liver	-
18AA_O	Oldřišov	Opava	2016	AA	F	AD	Lungs, liver	-
19AF_O	Oldřišov	Opava	2016	AF	M	AD	Lungs, liver	-
20AA_O	Oldřišov	Opava	2016	AA	M	SUB	Lungs, liver	-
21AF_O	Oldřišov	Opava	2016	AF	M	AD	Lungs	-
22AA_O	Oldřišov	Opava	2016	AA	F	AD	Lungs	-

23AA_O	Oldřišov	Opava	2016	AA	M	SUB	Lungs	-
24AF_O	Oldřišov	Opava	2016	AF	M	AD	Lungs	-
25AA_O	Oldřišov	Opava	2016	AA	M	SUB	Lungs	-
26AA_O	Oldřišov	Opava	2016	AA	F	SUB	Lungs	-
27AA_O	Oldřišov	Opava	2016	AA	F	SUB	Lungs	-
29AA_O	Oldřišov	Opava	2016	AA	F	AD	Lungs	-
30AA_O	Oldřišov	Opava	2016	AA	M	SUB	Lungs	-
31AA_O	Oldřišov	Opava	2016	AA	F	AD	Lungs	-
32AF_O	Oldřišov	Opava	2016	AF	F	AD	Lungs	-
33AA_O	Oldřišov	Opava	2016	AA	F	SUB	Lungs	-
34AA_O	Oldřišov	Opava	2016	AA	F	AD	Lungs	-
35AA_O	Oldřišov	Opava	2016	AA	M	AD	Lungs	-
36AA_O	Oldřišov	Opava	2016	AA	F	AD	Lungs	-
37AA_O	Oldřišov	Opava	2016	AA	F	SUB	Lungs	-
38AF_O	Oldřišov	Opava	2016	AF	F	AD	Lungs	-

39AA_O	Oldřišov	Opava	2016	AA	-	-	Lungs	-
40AA_O	Oldřišov	Opava	2016	AA	M	AD	Lungs, liver, brain	KURV lungs, liver, brain
41AA_O	Oldřišov	Opava	2016	AA	M	SUB	Lungs, liver, spleen	-
42AA_O	Oldřišov	Opava	2016	AA	M	SUB	Lungs, liver, spleen	-
43AA_O	Oldřišov	Opava	2016	AA	M	AD	Lungs, liver, spleen	KURV spleen
44AA_O	Oldřišov	Opava	2016	AA	M	SUB	Lungs, spleen	-
45MA_O	Oldřišov	Opava	2016	MA	F	AD	Lungs	-
46AA_O	Oldřišov	Opava	2016	AA	F	AD	Lungs, spleen	-
47AF_O	Oldřišov	Opava	2016	AF	F	AD	Lungs	-
48AA_Ř	Oldřišov, sugar beet field between Oldřišov and Opava	Opava	2016	AA	M	SUB	Lungs, spleen	TULV spleen
49AA_Ř	Oldřišov, sugar beet field between Oldřišov and Opava	Opava	2016	AA	M	AD	Lungs, liver, spleen, brain	KURV lungs, liver, spleen, brain
50AF_Ř	Oldřišov, sugar beet field between Oldřišov and Opava	Opava	2016	AF	M	AD	Lungs, spleen	-
51AA_Ř	Oldřišov, sugar beet field between Oldřišov and Opava	Opava	2016	AA	M	SUB	Lungs	-
52AA_H	Opava, weed hill near the Hillova street	Opava	2016	AA	F	AD	Lungs, liver, spleen, brain	KURV lungs

								TULV liver
53AA_H	Opava, weed hill near the Hillova street	Opava	2016	AA	F	JUV	Lungs, spleen	-
54AA_H	Opava, weed hill near the Hillova street	Opava	2016	AA	F	AD	Lungs, spleen	-
55AA_H	Opava, weed hill near the Hillova street	Opava	2016	AA	M	AD	Lungs, liver, spleen	KURV spleen
56AA_H	Opava, weed hill near the Hillova street	Opava	2016	AA	F	AD	Lungs, liver, spleen, brain	TULV lungs, liver
1AA_V	Varnsdorf	Děčín	2018/2019	AA	-	-	Lungs, liver, kidneys, heart	-
3AA_V	Varnsdorf	Děčín	2018/2019	AA	-	-	Lungs, liver, kidneys, heart	-
4MA_V	Varnsdorf	Děčín	2018/2019	MA	-	-	Lungs, liver, kidneys, heart	-
6CG_V	Varnsdorf	Děčín	2018/2019	CG	-	-	Lungs, liver, kidneys, heart	-
9AF_V	Varnsdorf	Děčín	2018/2019	AF	-	-	Lungs, liver, kidneys, heart	-
1AA_VAR	Varnsdorf	Děčín	2018/2019	AA	-	-	Lungs	-
7AA_VAR	Varnsdorf	Děčín	2018/2019	AA	-	-	Lungs	-
12AA_VAR	Varnsdorf	Děčín	2018/2019	AA	-	-	Lungs	-
15AA_VAR	Varnsdorf	Děčín	2018/2019	AA	-	-	Lungs	-
01AF_VES	Vestec, Biocev	Praha-západ	2020	AF	M	AD	Lungs, liver, kidneys	-

02AF_VES	Vestec, Biocev	Praha-západ	2020	AF	M	AD	Lungs, liver, kidneys	-
03AF_VES	Vestec, Biocev	Praha-západ	2020	AF	M	AD	Lungs, liver, kidneys	-
04AF_VES	Vestec, Biocev	Praha-západ	2020	AF	M	AD	Lungs, liver, kidneys	-
05AF_VES	Vestec, Biocev	Praha-západ	2020	AF	M	AD	Lungs, liver, kidneys	-
06AF_VES	Vestec, Biocev	Praha-západ	2020	AF	M	AD	Lungs, liver, kidneys	-
01MI_VES	Vestec, Biocev	Praha-západ	2020	MA	M	AD	Lungs, liver, kidneys	TULV lungs, liver, kidneys
02MI_VES	Vestec, Biocev	Praha-západ	2020	MA	M	AD	Lungs, liver, kidneys	TULV lungs, liver, kidneys
03MI_VES	Vestec, Biocev	Praha-západ	2020	MA	M	AD	Lungs, liver, kidneys	TULV liver
04MI_VES	Vestec, Biocev	Praha-západ	2020	MA	M	AD	Lungs, liver, kidneys	TULV lungs
05MI_VES	Vestec, Biocev	Praha-západ	2020	MA	M	AD	Lungs, liver, kidneys	TULV lungs, liver, kidneys
06MI_VES	Vestec, Biocev	Praha-západ	2020	MA	M	AD	Lungs, liver, kidneys	TULV lungs, liver, kidneys
07MI_VES	Vestec, Biocev	Praha-západ	2020	MA	M	AD	Lungs, liver, kidneys	TULV lungs, liver, kidneys
08MI_VES	Vestec, Biocev	Praha-západ	2020	MA	M	AD	Lungs, liver, kidneys	TULV lungs, liver, kidneys

09MI_VES	Vestec, Biocev	Praha-západ	2020	MA	M	AD	Lungs, liver, kidneys	TULV<br>lungs, liver, kidneys
09AF_VES	Vestec, Biocev	Praha-západ	2020	AF	M	AD	Lungs	-
11AS_VES	Vestec, Biocev	Praha-západ	2020	AS	M	AD	Lungs, liver, kidneys	-
12AF_VES	Vestec, Biocev	Praha-západ	2020	AF	M	AD	Lungs, liver, kidneys	TULV<br>lungs
1AS_VES	Vestec, Biocev	Praha-západ	2020	AS	F	AD	Lungs	-
2AS_VES	Vestec, Biocev	Praha-západ	2020	AS	F	AD	Lungs	-
1MIC_VES	Vestec, Biocev	Praha-západ	2020	MA	F	AD	Lungs, liver, kidneys	TULV<br>lungs, kidneys
2MIC_VES	Vestec, Biocev	Praha-západ	2020	MA	F	AD	Lungs, liver, kidneys	TULV<br>lungs, liver, kidneys
4MIC_VES	Vestec, Biocev	Praha-západ	2020	MA	F	AD	Lungs, liver, kidneys	TULV<br>lungs
5MIC_VES	Vestec, Biocev	Praha-západ	2020	MA	F	AD	Lungs, liver, kidneys	TULV<br>lungs
07AF_VES	Vestec, near the Shell gas station	Praha-západ	2020	AF	F	AD	Lungs, liver, kidneys	-
08AS_VES	Vestec, near the Shell gas station	Praha-západ	2020	AS	M	AD	Lungs, liver, kidneys	TULV<br>liver
3MIC_VES	Vestec, near the Shell gas station	Praha-západ	2020	MA	F	AD	Lungs	-
6MIC_VES	Dolní Břežany	Praha-západ	2020	MA	F	AD	Lungs, liver, kidneys	TULV<br>lungs

14AS_VES	Dolní Břežany	Praha-západ	2020	AS	F	AD	Lungs, liver, kidneys	TULV<br>lungs
6AS_VES	Dolní Břežany	Praha-západ	2020	AS	F	AD	Lungs	-
7MIC_VES	Dolní Břežany	Praha-západ	2020	MA	F	AD	Lungs, liver, kidneys	TULV<br>lungs, liver, kidneys
23934SM_VLT	Vltava	České Budějovice	2017	SM	-	-	Lungs, liver, kidneys, brain	-
5SA	Biology Centre CAS	České Budějovice	2020	SA	-	-	Lungs, liver, brain	SWSV<br>Lungs, liver, brain
7SA	Zbytiny – Koryto	Prachatice	2018	SA	-	-	Lungs, liver, kidneys, spleen, brain, heart	SWSV<br>heart
1CL	Semtěš	Karlovy Vary	2019	CL	-	-	Lungs, liver, heart	
2CS	Volenice	Strakonice	2019	CS	-	-	Lungs, brain, heart	-
2NF_LUŽ	Lužnice, field station U Zahradníků no. 92	Jindřichův Hradec	2018	NF	-	-	Lungs	-
9NF_LUŽ	Lužnice, field station U Zahradníků no. 92	Jindřichův Hradec	2018	NF	-	-	Lungs	-
3CS	Hoděmyšl	Příbram	2019	CS	-	-	Lungs, brain, heart	
4SA	Podmokly	Plzeň-sever	2019	SA	-	-	Lungs, brain, heart	ASIV<br>Lungs, brain, heart
2SA_VAR	Varnsdorf	Děčín	2018	SA	-	-	Lungs, liver, kidneys, heart	-

Supplementary Table S2. Details of the L segment sequences used for the phylogenetic analyses. Sequences obtained in this study are highlighted in bold. AMRV – Amur virus ; ASIV – Asikkala virus; DOBV – Dobrava virus, KURV – Kurkino virus; SAAV – Saaremaa virus; SOCV – Sochi virus, HTNV – Hantaan virus; PUUV – Puumala virus; SEOV – Seoul virus; SWSV – Seewis virus; TATV - Tatenale virus; TULV – Tula virus*;*

**Orthohantavirus**	**L segment GenBank Accession Number**	**Origin**	**Species of the host**	**Tissue**
AMRV	JX119008	China, Jilin Changbai	*Apodemus peninsulae*	n.a.
ASIV	NC043068	Czech Republic, Beskydy	*Sorex minutus*	n.a.
	C880349	Germany	*S. minutus*	
	**ON243817**	**Czech Republic, Podmokly**	***Sorex araneus* 4SA**	**lungs, heart**
DOBV	KF039740	Turkey, Istanbul	*Homo sapiens*	serum
	KF177176	Serbia, Ravanica	*Glis glis*	n.a.
	KF425497	Serbia, Tara Mt. tourist complex	*Apodemus flavicollis*	n.a.
	KT315630	Turkey, Igneada Region	*A. flavicollis*	lungs
	KT315631	Turkey, Igneada Region	*A. flavicollis*	lungs
	KY649165	Serbia	*H. sapiens*	
	MK605641	Czech Republic, Domašov nad Bystřicí	*H. sapiens*	HFRS serum
	MK605649	Czech Republic, Ostrava	*H. sapiens*	HFRS serum
	MK605651	Czech Republic, Karolinka	*H. sapiens*	HFRS serum
	MK605659	Czech Republic, Dolni Netcice	*H. sapiens*	HFRS serum

	MK605660	Czech Republic, Nedasov	*H. sapiens*	HFRS serum
	MK605664	Czech Republic, Jindřichov u Šumperka	*H. sapiens*	HFRS serum
	MK605679	Czech Republic, Čeladná	*A. flavicollis*	lungs
KURV	KF536031	Germany, Stockelsdorf	*H. sapiens*	HFRS serum
	KF536033	Germany, Neuruppin	*H. sapiens*	HFRS serum
	KF536035	Germany, Richtenberg	*H. sapiens*	HFRS serum
	KJ182938	Germany	*Apodemus agrarius*	lungs
	MK605642	Czech Republic, Josefovice	*H. sapiens*	HFRS serum
	MK605644	Czech Republic, Velka Kras	*H. sapiens*	HFRS serum
	MK605646	Czech Republic, Cernotin	*H. sapiens*	HFRS serum
	MK605647	Czech Republic, Ostrava	*H. sapiens*	HFRS serum
	MK605655	Czech Republic, Klimkovice	*H. sapiens*	HFRS serum
	MK605656	Czech Republic, Bohuslavice u Hlučína	*H. sapiens*	HFRS serum
	MK605661	Czech Republic, Velké Heraltice	*H. sapiens*	HFRS serum
	MK605665	Czech Republic, Šenov u Nového Jičína	*H. sapiens*	HFRS serum
	MK605680	Czech Republic, Petrovice	*A. agrarius*	lungs
	**ON243806**	**Czech Republic, Oldřišov**	***A. agrarius* 7AA**	**liver**

	**ON243807**	**Czech Republic, Oldřišov**	***A. agrarius* 10AA**	**liver**
	**ON243808**	**Czech Republic, Oldřišov**	***A. agrarius* 40AA**	**lungs, liver, brain**
	**ON243809**	**Czech Republic, Oldřišov**	***A. agrarius* 43AA**	**spleen**
	**ON243810**	**Czech Republic, Oldřišov, sugar beet field**	***A. agrarius* 49AA**	**lungs, liver, spleen, brain**
	**ON243811**	**Czech Republic, Opava, weed hill near the Hillova street**	***A. agrarius* 55AA**	**spleen**
	**ON243813**	**Czech Republic, České Budějovice, Borek**	***Apodemus sylvaticus* 23721AS**	**lungs**
	**ON243812**	**Czech Republic, České Budějovice, Borek**	***Microtus arvalis* 23720MA**	**spleen, brain**
	**ON243814**	**Czech Republic, České Budějovice, Borek**	***M. arvalis* 23723MA**	**spleen**
SAAV	AJ410618	Estonia, Saaremaa Island	*A. agrarius*	n.a.
SOCV	KM192209	Russia, Krasnodar Region	*Apodemus ponticus*	n.a.
	KU529945	Russia	*H. sapiens*	HFRS serum
HTNV	EU837270	China, Guizhou	*H. sapiens*	Vero E6 cells
PUUV	KX815401	Poland, Telesnica Oszwarowa	*Clethrionomys glareolus*	lungs
SEOV	KX064268	France, Turckheim	*H. sapiens*	serum
SWSV	JQ425313	Czech Republic, České Budějovice	*S. araneus*	n.a.
	JQ425317	Slovakia, Košice	*S. araneus*	n.a.

	KF974361	Japan, Hokkaido	*Sorex caecutiens*	lungs
	MT560038	Russia, Tomsk Oblast	*S. araneus*	lungs
	KY651062	Finland	*S. araneus*	lungs
	**ON243815**	**Czech Republic, České Budějovice, Biology Centre CAS**	***S. araneus* 5SA**	**lungs, liver, brain**
	**ON243816**	**Czech Republic, Zbytiny - Koryto**	***S. araneus* 7SA**	**heart**
TATV	JX316008	United Kingdom	*Microtus agrestis*	lungs
TULV	FJ495100	Slovenia, Sestrze	*M. agrestis*	lungs
	FJ495101	Slovenia, Hodos	*M. arvalis*	lungs
	FJ495102	Slovenia, Sred ob Dravi	*Microtus subterraneus*	lungs
	HQ728459	Germany	*Arvicola amphibius*	lungs
	HQ728464	Germany	*M. arvalis*	lungs
	KC522413	Czech Republic, Opava	*H. sapiens*	plasma
	KU297981	France, Chevru	*H. sapiens*	serum
	MK073348	Czech Republic	*M. arvalis*	lungs
	MK073356	Czech Republic	*M. arvalis*	lungs
	MK073366	Czech Republic	*M. arvalis*	lungs
	MK073367	Czech Republic	*M. arvalis*	lungs

	MK386155	Czech Republic	*M. arvalis*	lungs
	MK386156	Czech Republic	*M. arvalis*	lungs
	MK605685	Czech Republic, Beskydy	*M. agrestis*	lungs
	MT514295	Germany	*M. arvalis*	lungs
	MT514299	Germany	*M. arvalis*	lungs
	**ON243799**	**Czech Republic, Oldřišov**	***A. agrarius*** **9AA**	**liver**
	**ON243800**	**Czech Republic, Oldřišov**	***A. agrarius*** **15AA**	**liver**
	**ON243801**	**Czech Republic, Oldřišov, sugar beet field**	***A. agrarius*** **48AA**	**spleen**
	**ON243805**	**Czech Republic, Opava, weed hill near the Hillova street**	***A. agrarius*** **52AA**	**liver**
	**ON243802**	**Czech Republic, Opava, weed hill near the Hillova street**	***A. agrarius*** **56AA**	**lungs**
	**ON243792**	**Czech Republic, Vestec, Biocev**	***A. flavicollis*** **12AF**	**lungs**
	**ON243788**	**Czech Republic, Vestec, near the Shell gas station**	***A. sylvaticus*** **8AS**	**liver**
	**ON243793**	**Czech Republic, Dolní Břežany**	***A. sylvaticus*** **14AS**	**lungs**
	**ON243780**	**Czech Republic, Lužnice**	***C. glareolus*** **3CG**	**lungs**
	**ON243781**	**Czech Republic, Lužnice**	***C. glareolus*** **4CG**	**lungs**
	**ON243803**	**Czech Republic, České Budějovice, Borek**	***M. arvalis*** **23723MA**	**lungs**

	**ON243804**	**Czech Republic, České Budějovice, Borek**	***M. arvalis*** **23729MA**	**lungs, kidneys**
	**ON243778**	**Czech Republic, Vestec, Biocev**	***M. arvalis*** **1MI**	**lungs, liver, kidneys**
	**ON243779**	**Czech Republic, Vestec, Biocev**	***M. arvalis*** **2MI**	**lungs, liver, kidneys**
	**ON243782**	**Czech Republic, Vestec, Biocev**	***M. arvalis*** **3MI**	**liver**
	**ON243783**	**Czech Republic, Vestec, Biocev**	***M. arvalis*** **4MI**	**lungs**
	**ON243784**	**Czech Republic, Vestec, Biocev**	***M. arvalis*** **5MI**	**lungs, liver, kidneys**
	**ON243785**	**Czech Republic, Vestec, Biocev**	***M. arvalis*** **6MI**	**liver**
	**ON243786**	**Czech Republic, Vestec, Biocev**	***M. arvalis*** **7MI**	**liver**
	**ON243787**	**Czech Republic, Vestec, Biocev**	***M. arvalis*** **7MI**	**lungs, kidneys**
	**ON243789**	**Czech Republic, Vestec, Biocev**	***M. arvalis*** **8MI**	**liver**
	**ON243790**	**Czech Republic, Vestec, Biocev**	***M. arvalis*** **8MI**	**lungs, kidneys**
	**ON243791**	**Czech Republic, Vestec, Biocev**	***M. arvalis*** **9MI**	**lungs, liver, kidneys**
	**ON243794**	**Czech Republic, Vestec, Biocev**	***M. arvalis*** **1MIC**	**lungs, kidneys**
	**ON243795**	**Czech Republic, Vestec, Biocev**	***M. arvalis*** **2MIC**	**lungs, liver, kidneys**
	**ON243796**	**Czech Republic, Vestec, Biocev**	***M. arvalis*** **5MIC**	**lungs**

	**ON243797**	**Czech Republic, Dolní Břežany**	***M. arvalis*** **6MIC**	**lungs**
	**ON243798**	**Czech Republic, Dolní Břežany**	***M. arvalis*** **7MIC**	**lungs, liver, kidneys**

Supplementary Table S3. Details of the M segment sequences used for the phylogenetic analyses. Sequences obtained in this study are highlighted in bold.). DOBV – Dobrava virus, SAAV – Saaremaa virus; SOCV – Sochi virus, HTNV – Hantaan virus; SWSV – Seewis virus; TULV – Tula virus*;*

**Orthohantavirus**	**M segment GenBank Accession Number**	**Origin**	**Species of the Host**	**Tissue**
Dobrava-Belgrade orthohantavirus (virus not specified)	AY961616	Slovakia	*Apodemus agrarius*	Vero E6 cells
	EU188453	Russia, Lipetsk	*A. agrarius*	Vero E6 cells
	JQ026205	Germany, Greifswald	*Apodemus flavicollis*	lungs
	KF536034	Germany, Neuruppin	*Homo sapiens*	HFRS serum
	**ON243777**	**Czech Republic, Oldřišov**	***A. agrarius* 40AA**	**lungs**
DOBV	AY168577	Slovakia, East Slovakia	*A. flavicollis*	lungs
	KT885042	Slovenia, Stamforrad	*A. flavicollis*	Vero E6 cells
SAAV	AJ616855	Denmark, Lolland	*A. agrarius*	lungs
	FN813293	Croatia, Migalovci	*A. flavicollis*	lungs
SOCV	JF920149	Russia	*H. sapiens*	HFRS serum
HTNV	KU207185	South Korea	*A. agrarius*	lungs
SWSV	GQ293139	Hungary	*Sorex araneus*	liver
	JX990967	Poland, Boginia	*S. araneus*	lungs
	KM201415	Russia	*Sorex caecutiens*	lungs
	MT951408	Russia	*S. caecutiens*	n.a.
TULV	MH649271	Turkey, Palandoken	*Microtus obscurus*	lungs
	MK386142	Germany	*Microtus arvalis*	lungs

	MK386143	Czech Republic	*M. arvalis*	lungs
	MK386144	Czech Republic	*M. arvalis*	lungs
	MK386148	Czech Republic	*M. arvalis*	lungs
	MT514285	Germany	*M. arvalis*	lungs
	**ON653433**	**Czech Republic, České Budějovice, Švábův Hrádek**	***A. flavicollis* 10AF**	**lungs**
	**ON653442**	**Czech Republic, Vestec, near the Shell gas station**	***Apodemus sylvaticus* 8AS**	**liver**
	**ON653435**	**Czech Republic, Dolní Břežany**	***A. sylvaticus* 14AS**	**lungs**
	**ON653441**	**Czech Republic, České Budějovice, Borek**	***M. arvalis* 23723MA**	**lungs**
	**ON653425**	**Czech Republic, Vestec, Biocev**	***M. arvalis* 1MI**	**lungs, liver, kidneys**
	**ON653426**	**Czech Republic, Vestec, Biocev**	***M. arvalis* 2MI**	**lungs**
	**ON653427**	**Czech Republic, Vestec, Biocev**	***M. arvalis* 4MI**	**lungs**
	**ON653428**	**Czech Republic, Vestec, Biocev**	***M. arvalis* 5MI**	**lungs**
	**ON653429**	**Czech Republic, Vestec, Biocev**	***M. arvalis* 6MI**	**lungs, liver, kidneys**
	**ON653430**	**Czech Republic, Vestec, Biocev**	***M. arvalis* 7MI**	**lungs, liver, kidneys**
	**ON653431**	**Czech Republic, Vestec, Biocev**	***M. arvalis* 8MI**	**lungs, liver, kidneys**
	**ON653432**	**Czech Republic, Vestec, Biocev**	***M. arvalis* 9MI**	**lungs, liver**
	**ON653436**	**Czech Republic, Vestec, Biocev**	***M. arvalis* 1MIC**	**lungs, liver**
	**ON653437**	**Czech Republic, Vestec, Biocev**	***M. arvalis* 2MIC**	**lungs, liver**
	**ON653438**	**Czech Republic, Vestec, Biocev**	***M. arvalis* 4MIC**	**lungs**

	**ON653439**	**Czech Republic, Vestec, Biocev**	***M. arvalis* 6MIC**	**lungs**
	**ON653440**	**Czech Republic, Dolní Břežany**	***M. arvalis* 7MIC**	**lungs, liver, kidneys**
	**ON653434**	**Czech Republic, Vestec, Biocev**	***A. flavicollis* 12AF**	**lungs**

Supplementary table S4. Tissue tropism – prevalence of detected hantaviruses in different host tissues. TULV – Tula virus; KURV – Kurkino virus; SWSV – Seewis virus; ASIV – Asikkala virus

**Host species**	**Orthohantavirus**	**Lungs**	**Liver**	**Kidneys**	**Spleen**	**Brain**	**Heart**
Microtus arvalis	TULV	100% (17/17)	66.7% (10/15)	64.7% (11/17)	n.a.	n.a.	n.a.
	KURV	0% (0/2)	100% (1/1)	0% (0/2)	100% (2/2)	100% (1/1)	0% (0/1)
Clethrionomys glareolus	TULV	100% (2/2)	n.a.	n.a.	n.a.	n.a.	n.a.
	KURV	n.a.	n.a.	n.a.	n.a.	n.a.	n.a.
Apodemus agrarius	TULV	20.0% (1/5)	100% (4/4)	n.a.	20.0% (1/5)	0% (0/2)	n.a.
	KURV	42.9% (3/7)	57.1% (4/7)	n.a.	50.0% (3/6)	66.7% (2/3)	n.a.
Apodemus flavicollis	TULV	100% (2/2)	0% (0/2)	0% (0/2)	n.a.	n.a.	n.a.
	KURV	0% (0/2)	0% (0/2)	0% (0/2)	n.a.	n.a.	n.a.
Apodemus sylvaticus	TULV	50.0% (1/2)	50.0% (1/2)	0% (0/2)	n.a.	n.a.	n.a.
	KURV	100% (2/2)	n.a.	0% (0/2)	n.a.	0% (0/1)	0% (0/1)
Sorex araneus	SWSV	50.0% (1/2)	50.0% (1/2)	0% (0/1)	0% (0/1)	50.0% (1/2)	100% (1/1)
	ASIV	100% (1/1)	n.a.	n.a.	n.a.	100% (1/1)	100% (1/1)
